# A noise-robust classification method for cryo-ET subtomograms with out-of-distribution detection

**DOI:** 10.1093/bioinformatics/btaf274

**Published:** 2025-05-13

**Authors:** Wenjia Meng, Xueshi Yu, Tingting Zhang, Renmin Han

**Affiliations:** School of Software, Shandong University, Jinan 250101, China; School of Software, Shandong University, Jinan 250101, China; School of Software, Shandong University, Jinan 250101, China; College of Medical Information and Engineering, Ningxia Medical University, Yinchuan 750004, China; Research Center for Mathematics and Interdisciplinary Sciences (Ministry of Education Frontiers Science Center for Nonlinear Expectations), Shandong University, Qingdao 266237, China

## Abstract

**Motivation:**

Cryogenic electron tomography (cryo-ET) enables high-resolution 3D reconstruction of biological samples, with accurate subtomogram classification critical for structural analysis. However, current subtomogram classification methods often struggle with out-of-distribution (OOD) data issue, causing misclassification and mismatched structures.

**Results:**

To solve this problem, we propose a unified subtomogram classification framework that incorporates OOD detection to distinguish unknown (OOD) from known (in-distribution, ID) classes and predict labels for ID data, thereby enhancing existing subtomogram classification methods. Within this framework, we develop a noise-robust classification method that integrates a 3D discrete wavelet transform-based encoder to reduce high-frequency noise and extract robust features. Additionally, we incorporate a Mahalanobis distance-based OOD detector with a reliable metric for 3D subtomograms and introduce an adaptive classifier that adjusts to accommodate datasets of varying scales. The experimental and visualization results demonstrate that our noise-robust method improves subtomogram classification accuracy and effectively models features while enhancing OOD detection.

**Availability and implementation:**

Our code is available at https://github.com/yxs1137/Subtomo-Classification-with-OOD.git. The real data used in this study can be accessed through CryoET Data Portal.

## 1 Introduction

Cryogenic electron tomography (cryo-ET) is an important imaging technique used to reconstruct high-resolution 3D volumes of biological macromolecules and cell samples. Cryo-ET serves as the foundation for downstream tasks such as structure detection of interest (Martinez-Sanchez *et al.* 2020), subtomogram classification (Xu *et al.* 2017), and the analysis of fundamental life processes at the subcellular level (Nogales and Mahamid 2024). Among these tasks, subtomogram classification (Förster *et al.* 2008) plays an important role. It aims to predict the classification labels of subtomograms that contain only a single macromolecule. Accurate subtomogram classification of the structures not only enhances the performance of downstream steps, but also aids researchers in gaining a deeper understanding of life processes at the subcellular level.

**Figure 1.**
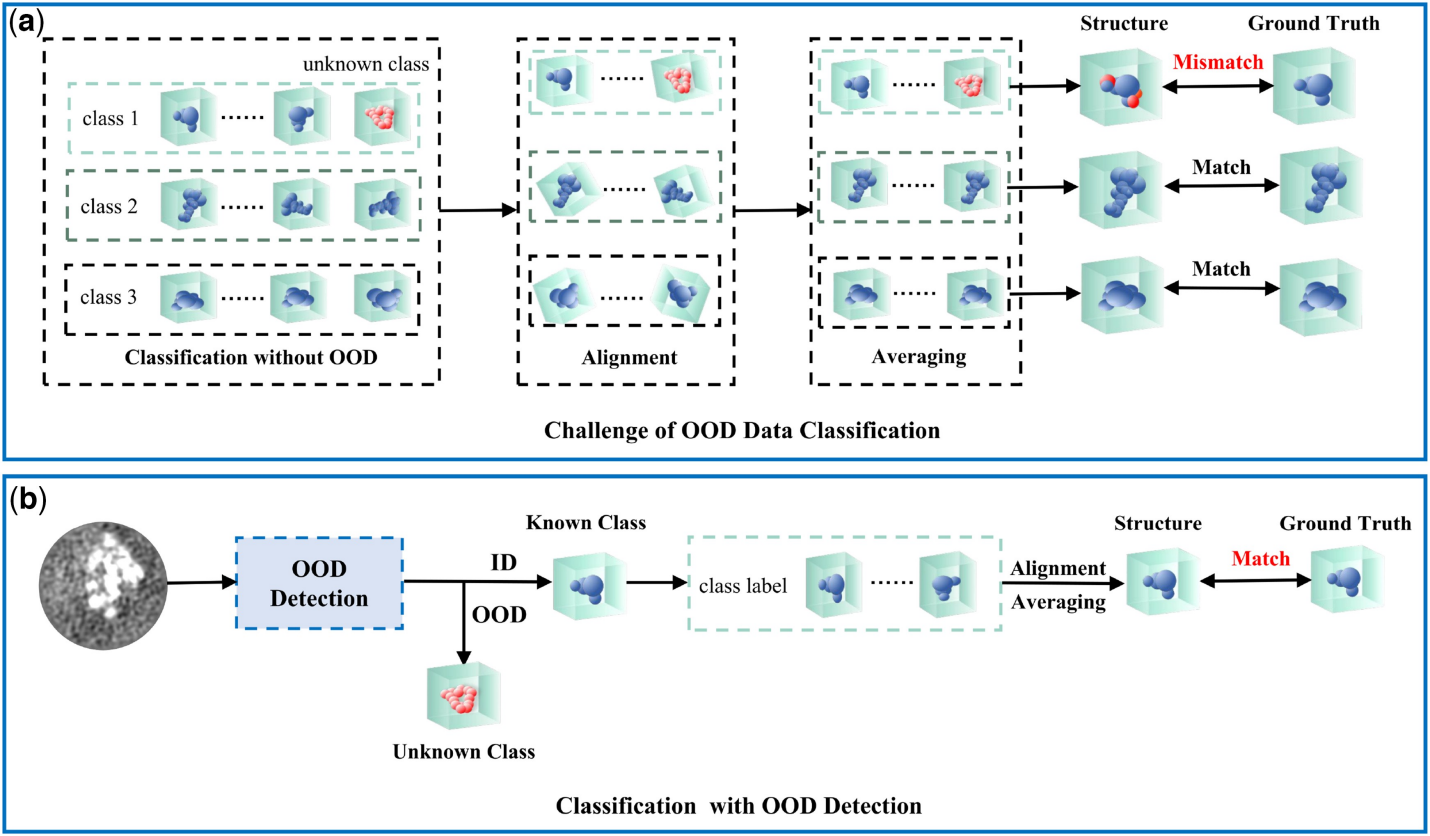
(a) Challenge of OOD data classification: misclassification of OOD data by existing methods leads to the structural mismatch with the ground truth. (b) Classification with OOD detection: OOD detection discards unknown-class data, enabling downstream tasks to achieve accurate structures.

Subtomogram classification methods can be broadly categorized into four types: template matching methods, supervised learning-based methods, unsupervised learning-based methods, and few-shot learning-based methods. Template matching methods (Frangakis *et al.* 2002, Förster *et al.* 2008) identify potential particles and their positions by scanning the entire tomogram with a low-resolution target macromolecular template and using high cross-correlation scores. Supervised learning-based methods (Xu *et al.* 2017, Che *et al.* 2018) train classification models on large amounts of labeled data to achieve accurate subtomogram classification. Unsupervised learning-based methods (Zeng and Xu 2020, Zeng *et al.* 2023) do not rely on fully labeled data, but instead perform classification and clustering by learning the intrinsic structure of the data. Few-shot learning-based methods (Yu *et al.* 2021, 2024) enable classification models to quickly adapt to new tasks with only one or a few labeled subtomograms. However, these above subtomogram classification methods fail to address the challenge of out-of-distribution (OOD) data, as illustrated in Fig. 1a. This challenge lies in that existing methods misclassify the unknown class into one of the known classes. Based on misclassified samples, the subtomogram alignment and averaging process (Bartesaghi and Subramaniam 2009) results in a 3D reconstructed structure that mismatches the ground truth, leading to misleading outcomes in subsequent structural and functional analysis.

To address this challenge, we propose a subtomogram classification framework with the OOD detection, which distinguishes unknown (OOD) from known (ID) classes and predicts labels for ID data to enable accurate alignment, averaging, and 3D reconstruction, effectively handling ID and OOD data (illustrated in Fig. 1b). Within the proposed framework, we introduce a noise-robust subtomogram classification method that integrates a 3D discrete wavelet transform-based encoder with a Mahalanobis distance-based OOD detector to robustly classify the subtomogram data. The 3D discrete wavelet transform-based encoder is designed to extract robust features from subtomogram data, effectively reducing high-frequency noise and enhancing feature reliability. Building on these features, the Mahalanobis distance-based OOD detector leverages a reliable metric, making it particularly well-suited for 3D subtomogram data. To accommodate datasets with varying scales, we further propose an adaptive classifier that adjusts the inner product operation between feature vectors and classifier weights. Extensive experiments conducted on both simulated and real datasets validate the effectiveness of the proposed method, demonstrating its robustness in OOD detection and classification tasks.

## 2 Proposed method

In this section, we address the challenge of subtomogram classification with OOD data by proposing a framework that integrates OOD detection to distinguish OOD from ID data and accurately predict ID class labels. Within the proposed framework, we then present a robust subtomogram classification method to effectively handle the noisy data.

### 2.1 Proposed subtomogram classification framework with OOD detection

In this section, we first introduce the problem formulation of subtomogram classification with OOD detection, then detail the workflow of the proposed framework.

*Problem formulation of subtomogram classification with OOD detection.* In subtomogram classification with OOD detection, the dataset D consists of the ID dataset $D_{\text{ID}}$ and the OOD dataset $D_{\text{OOD}}$:


(1) $$D=D_{\text{ID}}\cup D_{\text{OOD}},$$

(2) $$D_{\text{ID}}={\left\{(x_{i},y_{i})\right\}}_{i=1}^{N_{\text{ID}}},\;D_{\text{OOD}}={\left\{(x_{j},y_{j})\right\}}_{j=1}^{N_{\text{OOD}}}.$$

Firstly, we perform OOD detection to identify whether a data *x* belongs to the ID or OOD category:


(3) $$g(x)=\{\begin{array}{cc} 1, & \text{ID},\\ 0, & \text{OOD}, \end{array}$$

where x∈D represents the subtomogram data, with g(x)=1 indicating that *x* is ID and g(x)=0 indicating it is OOD. Then, we perform classification on subtomograms identified as ID. Let $X\in R^{3}$ and Y={1,2,…,C} represent the subtomogram space and the label space of the ID macromolecule, respectively. Let $P_{XY}$ denote the joint data distribution. We can train a classification model:


(4) $$F:X\to R^{\left|Y\right|},$$

which is used to predict the label of an input subtomogram.

**Figure 2.**
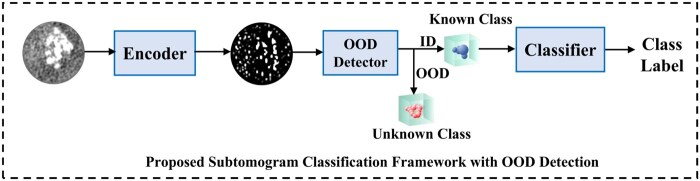
The proposed subtomogram classification framework with OOD detection. In the proposed framework, a 3D subtomogram is input into an encoder to extract features, followed by OOD detection to identify ID or OOD. For ID data, the classifier predicts its class label.

*Workflow for proposed framework.*  Figure 2 shows the workflow of the subtomogram classification framework with OOD detection. First, it determines whether a subtomogram is OOD or ID and then classifies it. During inference, the subtomogram *x* is passed through a feature extractor, followed by an OOD detector. If *x* is OOD, it is discarded; if ID, its feature map is classified to predict $\hat{y}\in Y$. This framework integrates OOD detection and can be applied to existing classification methods.

### 2.2 Proposed noise-robust subtomogram classification method with OOD detection

To further improve the classification methodology within the proposed framework, we introduce a noise-robust subtomogram classification method with OOD detection, which utilizes a 3D discrete wavelet transform to reduce high-frequency noise during feature extraction process.

#### 2.2.1 Training process and inference process

**Figure 3.**
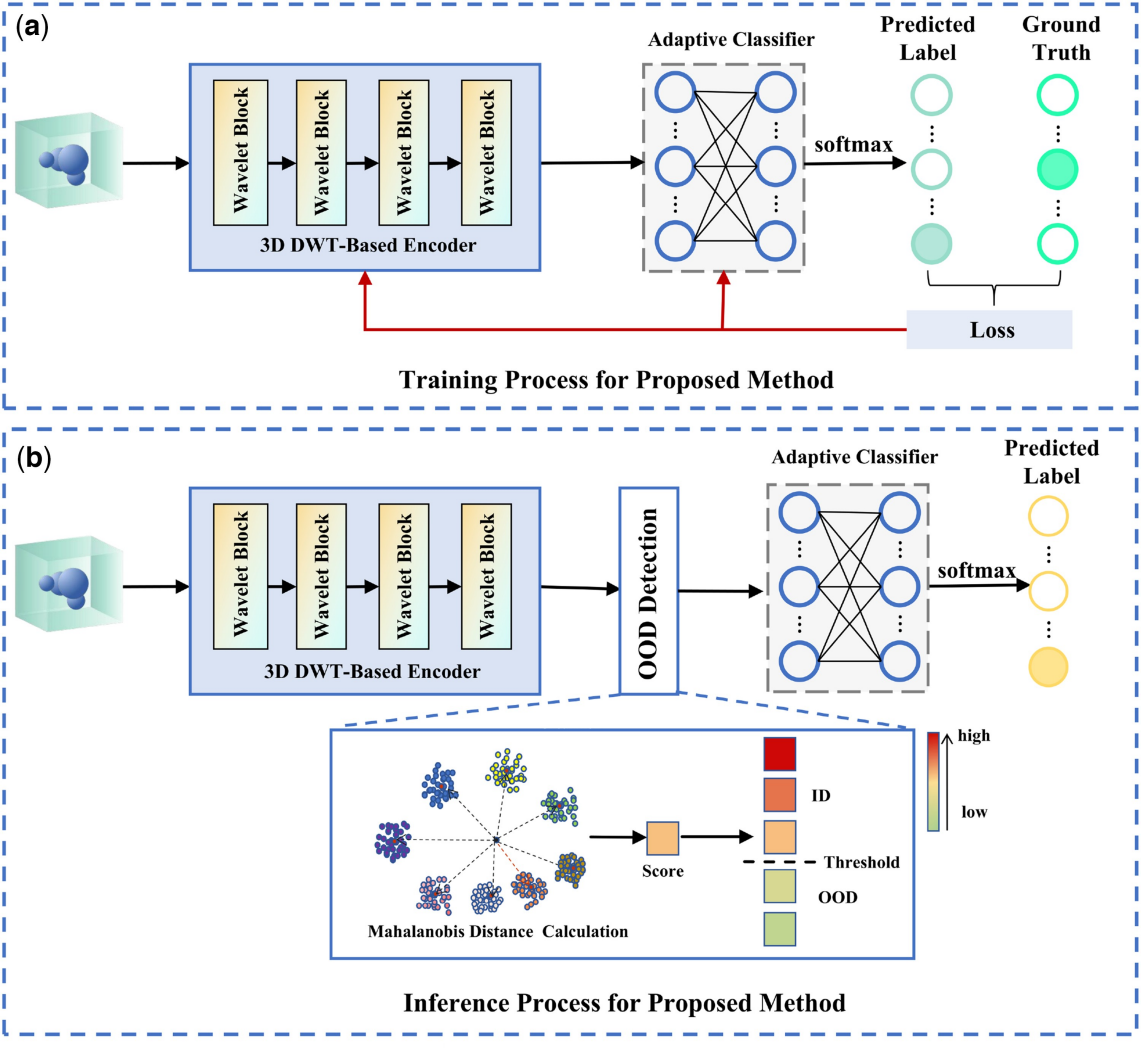
Processes for proposed noise-robust subtomogram classification method with OOD detection. (a) Training process. (b) Inference process.

In this section, we provide a detailed introduction of the training and inference processes of the proposed method, as illustrated in Fig. 3.

To clearly clarify these processes, we outline three key components of the proposed method: a 3D DWT-based encoder, an Mahalanobis distance-based OOD detector, and an adaptive classifier. During the training phase (Fig. 3a), the proposed noise-robust subtomogram classification method first encodes raw subtomogram data from the training dataset using the 3D DWT-based encoder. We use random rotation-based data augmentation to robustly learn rotation-invariant features. The extracted features are then passed to an adaptive classifier, comprising a fully connected layer and a softmax operation, to predict class labels. Cross-entropy loss is used to optimize the classification method throughout the training process. In the inference phase (Fig. 3b), the 3D DWT-based encoder is first used to extract features from 3D subtomograms in the test datasets. The Mahalanobis distance-based OOD detector subsequently determines whether the subtomogram belongs to ID or OOD. Subtomograms identified as OOD are discarded, whereas those classified as ID are passed to the classifier for final class label prediction.

#### 2.2.2 3D DWT-based encoder

**Figure 4.**
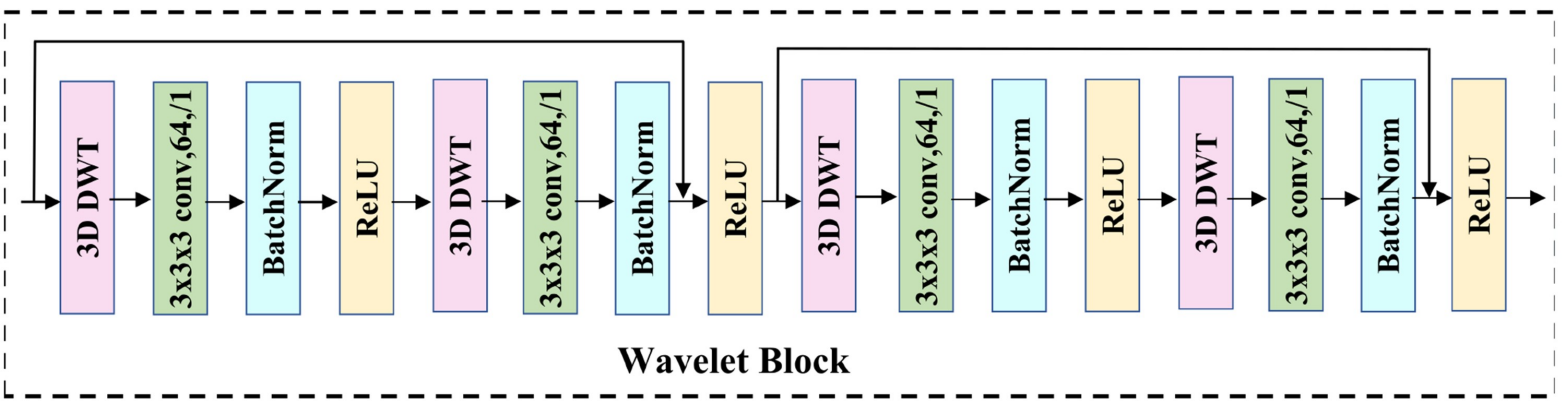
The detailed structure of our wavelet block, composed of four main modules: 3D discrete wavelet transform (3D DWT), convolution, BatchNorm, and ReLU. “3x3x3 conv, 64,/1” refers to a convolutional layer with a 3x3x3 kernel, 64 output channels, and a stride of 1.

This section describes the 3D DWT-based encoder, which improves noise robustness by filtering high-frequency noise. The encoder consists of four wavelet blocks (Fig. 4), each with 3D DWT, convolution, BatchNorm, and ReLU. The process includes two iterations: 3D DWT filters and downsamples the data, followed by convolution, BatchNorm, and ReLU. In the second iteration, another 3D DWT, convolution, and BatchNorm are applied. Finally, the features are fused with the residuals and refined by ReLU.

*3D discrete wavelet transform.* The 3D DWT module is a key component of the proposed encoder. We first introduce the discrete wavelet transform and then explain the 3D DWT in the context of subtomogram classification.

The discrete wavelet transform (DWT) efficiently decomposes a signal into different frequency components, analyzing their distribution in time or space (Mallat 1989). For 1D data *x*, it separates into low-frequency $x_{\mathrm{low}}$ and high-frequency $x_{\mathrm{high}}$ components:


(5) $$x=\{x^{i}|0\leq i\leq m-1\},$$

(6) $$x_{\mathrm{low}}=\{x_{\mathrm{low}}^{j}|0\leq j\leq \left\lfloor \frac{m}{2}\right\rfloor -1\},$$

(7) $$x_{\mathrm{high}}=\{x_{\mathrm{high}}^{j}|0\leq j\leq \left\lfloor \frac{m}{2}\right\rfloor -1\},$$

where *m* denotes the length of *x* and ⌊·⌋ denotes the operation of rounding down. The $x_{\mathrm{low}}^{j}$ and $x_{\mathrm{high}}^{j}$ components are decomposed using the low-pass filter $l=\{l_{k}|k\in Z\}$ and the high-pass filter $h=\{h_{k}|k\in Z\}$, where Z represents the set of integers. The discrete wavelet transform is formulated by:


(8) $$x_{\mathrm{low}}^{j}=\sum_{i}x^{i}l_{i-2j},\;x_{\mathrm{high}}^{j}=\sum_{i}x^{i}h_{i-2j},$$

According to Equation (8), we formalize the DWT process as a matrix multiplication operation:


(9) $$x_{\mathrm{low}}=Lx,\;x_{\mathrm{high}}=Hx,$$

where both L and H are filter matrices with $\left\lfloor \frac{m}{2}\right\rfloor$ rows and *m* columns. Their formulations are as follows:


(10) $$\begin{array}{cc} L= & \left(\begin{array}{ccccccc} l_{0} & l_{1} & \cdots & & & & \\ \cdots & l_{-1} & l_{0} & l_{1} & \cdots & & \\ & & \cdots & l_{-1} & l_{0} & l_{1} & \cdots \\ & & & & & \cdots & \cdots \end{array}\right), \end{array}$$

(11) $$\begin{array}{cc} H= & \left(\begin{array}{ccccccc} h_{0} & h_{1} & \cdots & & & & \\ \cdots & h_{-1} & h_{0} & h_{1} & \cdots & & \\ & & \cdots & h_{-1} & h_{0} & h_{1} & \cdots \\ & & & & & \cdots & \cdots \end{array}\right). \end{array}$$

The matrices L and H each consist of $\left\lfloor \frac{m}{2}\right\rfloor$ row vectors, where the *j*th row vectors are defined as $L_{j}=\{l_{-2j},\ldots ,l_{m-1-2j}\}$ and $H_{j}=\{h_{-2j},\ldots ,h_{m-1-2j}\}$, respectively, with each row comprising *m* filter values (Li *et al.* 2021).

For 3D data $x\in R^{m_{H}\times m_{W}\times m_{D}}$, where $m_{H}$, $m_{W}$, and $m_{D}$ represent the height, width, and depth, respectively, the 3D discrete wavelet transform is performed by sequentially applying the 1D DWT along each dimension. The process begins with the second dimension (width), followed by the first dimension (height), and concludes with the third dimension (depth). The detailed 3D DWT operations are as follows:


(12) $$x_{\mathrm{lll}}=L{\left(LxL^{T}\right)}^{T_{1,3}},\;x_{\mathrm{llh}}=H{\left(LxL^{T}\right)}^{T_{1,3}},$$

(13) $$x_{\mathrm{lhl}}=L{\left(HxL^{T}\right)}^{T_{1,3}},\;x_{\mathrm{lhh}}=H{\left(HxL^{T}\right)}^{T_{1,3}},$$

(14) $$x_{\mathrm{hll}}=L{\left(LxH^{T}\right)}^{T_{1,3}},\;x_{\mathrm{hlh}}=H{\left(LxH^{T}\right)}^{T_{1,3}},$$

(15) $$x_{\mathrm{hhl}}=L{\left(HxH^{T}\right)}^{T_{1,3}},\;x_{\mathrm{hhh}}=H{\left(HxH^{T}\right)}^{T_{1,3}},$$

where $T_{1,3}$ denotes the transpose operation between the first and third dimensions of a 3D matrix, In the decomposed data, the subscripts indicate the DWT filter applied along the first (height), second (width), and third (depth) dimensions, with *l* and *h* representing the low-pass and high-pass filters, respectively. We retain the low-frequency component $x_{\mathrm{lll}}$ [Equation (12)] and discard the high-frequency components, thereby reducing high-frequency noise. This $x_{\mathrm{lll}}$ is subsequently used as the input for the next module in the wavelet block to enhance the method’s robustness against noise.

Utilizing the 3D discrete wavelet transform alongside additional modules (Figs 3 and 4), the 3D DWT-based encoder efficiently extracts robust features from the 3D subtomogram data *x*. The encoding process is defined as:


(16) z=E(x),

where E(·) represents the feature extraction operation performed by the 3D DWT-based encoder, *z* denotes the extracted feature.

#### 2.2.3 Mahalanobis distance-based OOD detector

In the inference process, given a test data *x*, we use the 3D DWT-based encoder to extract its feature z=E(x) and propose an OOD detector using Mahalanobis distance for OOD detection, as illustrated in Fig. 3b. This Mahalanobis distance-based OOD detector integrates covariance to capture the intrinsic correlations among features, providing a more reliable distance metric, especially for high-dimensional data analysis and OOD detection (Xiang *et al.* 2008).

During the OOD detection, we first calculate the Mahalanobis distance $d_{M}$ between feature *z* and the Gaussian distribution $G_{c}$, defined by the class prototype $\mu_{c}$ and the covariance Σ of the training samples (Mahalanobis 2018):


(17) $$d_{M}(z,G_{c})=\sqrt{{\left(z-\mu_{c}\right)}^{T}\Sigma^{-1}(z-\mu_{c})}.$$

where c∈{1,2,…,C} denotes the class label, $\Sigma^{-1}$ denotes the inverse of the matrix Σ, $\sqrt{\cdot }$ denotes the square root operation. The class prototype $\mu_{c}$ (mean) of *c*th class and the covariance matrix of training samples are defined as:


(18) $$\mu_{c}=\frac{1}{N_{c}}\sum_{i:y_{i}=c}z_{i},$$

(19) $$\Sigma =\frac{1}{N}\sum_{c}\sum_{i:y_{i}=c}(z_{i}-\mu_{c}){\left(z_{i}-\mu_{c}\right)}^{⊤},$$

where $N_{c}$ denotes the number of training samples with label *c*, and *N* represents the total number of training samples. Using the formulation in Equation (17), we compute the Mahalanobis distances for all classes ($d_{M}(z,G_{c}),c\in \{1,2,\ldots ,C\}$). Based on these distances, we define the confidence score S(z) as follows:


(20) $$S(z)=\underset{c}{\max}-{\left(d_{M}(z,G_{c})\right)}^{2}.$$

Using the score S(z) in Equation (20), we can determine whether a subtomogram comes from in-distribution or out-of-distribution:


(21) $$Q(z)=\{\begin{array}{cc} \text{ID}, & S(z)\geq \lambda ,\\ \text{OOD}, & S(z)<\lambda , \end{array},$$

where z=E(x) denote the feature of test subtomogram data *x* [see Equation (16)], λ denotes the threshold hyperparameter and is typically selected to ensure a high proportion of correctly classified ID data (e.g. 95%).

#### 2.2.4 Adaptive subtomogram classifier

Given a subtomogram data *x*, we extract its feature *z* using the 3D DWT-based encoder [Equation (16)]. During training, we classify *z* using the classifier. In the inference phase, we input *z* into the OOD detector to check if it is ID or OOD [Equation (21)]. If it is ID, we pass it to the classifier for label prediction. In this section, we propose an adaptive subtomogram classifier (see Fig. 3), designed to handle subtomogram datasets of varying scales.

The proposed subtomogram classifier comprises a single fully connected layer with weights:


(22) $$W=\{w_{1},\ldots ,w_{c},\ldots ,w_{C}\},$$

where $w_{c}$ represents the classifier weight for class label *c*. To enable its adaptability to subtomogram datasets of varying scales, we incorporate an adaptive inner product operation between the classifier weights $w_{c}$ and the feature vector *z*, thereby addressing both the small-scale real datasets and the large-scale simulated datasets. This adaptive classifier can be formulated as:


(23) $$CF(w_{c},z)=\{\begin{array}{cc} {\langle w_{c},z\rangle }_{\;\text{cos}\;}, & \text{large-scale}\;\text{dataset},\\ {\langle w_{c},z\rangle }_{t-\text{vMF}}, & \text{small-scale}\;\text{dataset}, \end{array}$$

where ${\langle w_{c},z\rangle }_{\;\text{cos}\;}$ and ${\langle w_{c},z\rangle }_{t-\text{vMF}}$ represent the inner products based on cosine similarity and t-vMF similarity (Kobayashi 2021), respectively. Previous studies (Zhang *et al.* 2019) show that cosine similarity-based classifiers perform well on large-scale datasets, while t-vMF similarity is better for small-scale datasets with high within-class variance, as it reduces variance by aligning feature vectors *z* with classifier weights $w_{c}$ of the ground truth class *c* (Kobayashi 2021).

Cosine similarity-based inner product and t-vMF similarity-based pseudo inner product in Equation (23) are defined as:


(24) $${\langle w_{c},z\rangle }_{\;\text{cos}\;}=||w_{c}||\;||z||\;\text{cos}(\theta ),$$

(25) $${\langle w_{c},z\rangle }_{t-\text{vMF}}=||w_{c}||\;||z||\phi (\text{cos}(\theta );\tau ),$$

where θ represents the angle between the feature vector *z* and the classifier weights $w_{c}$, ∥·∥ denotes the $L_{2}$ norm, cos(·) denotes the cosine similarity, ϕ(·;τ) represents the t-vMF similarity parameterized by τ:


(26) $$\phi (\text{cos}(\theta );\tau )=\frac{1+\text{cos}(\theta )}{1+\tau (1-\text{cos}(\theta ))}-1.$$

We then apply the softmax operation to the output of the classifier [Equation (23)] to obtain the probability that data *x* belongs to class *c*:


(27) $$P(y=c|x)=\frac{e^{CF(w_{c},z)}}{\sum_{j=1}^{C}e^{CF(w_{j},z)}},$$

where z=E(x) denotes the feature of data *x*, and *C* denotes the number of classes. Based on Equation (27), we can predict the class $\hat{y}$ of a subtomogram *x*:


(28) $$\hat{y}=\arg\underset{c}{\max}P(y=c|x).$$

We use the cross-entropy loss and minimize it to train the proposed subtomogram classification method:


(29) $$\mathrm{Loss}=-\frac{1}{N}\sum_{x_{i},y_{i}\in D}\;\text{log}\;P(y=y_{i}|x_{i}),$$

where D represents the training dataset and *N* denotes its size, $x_{i}$ is a sample in D, and $y_{i}$ is its ground truth label.

## 3 Experiments

In this section, we conduct experiments to evaluate the proposed method. We first describe the experimental setup. To assess the effectiveness of our framework, we compare existing methods with and without OOD detection. We also compare our noise-robust method with other state-of-the-art (SOTA) methods that include OOD detection. Additionally, we visually demonstrate the OOD detection capability using t-distributed Stochastic Neighbor Embedding (t-SNE) visualization and score distribution. Finally, we perform ablation experiments to validate the key modules.

### 3.1 Experimental setup

In this section, we introduce our experimental setup, including experimental details, dataset setting and evaluation metrics.

*Experimental details.* We used a 3D DWT-based ResNet18 as our encoder, with wavelet filters detailed in the Supplementary Material. For the Real dataset, we used a t-vMF similarity-based classifier with τ=64, while for SHREC19 and SHREC21, we used a cosine similarity-based classifier [see Equation (23)]. During training, we optimized the encoder for 100 epochs with a batch size of 64, using cross-entropy loss, an SGD optimizer with a learning rate of 0.1, momentum of 0.9, and weight decay of 0.0001. Inference used an OOD detection threshold of 95%.

**Figure 5.**
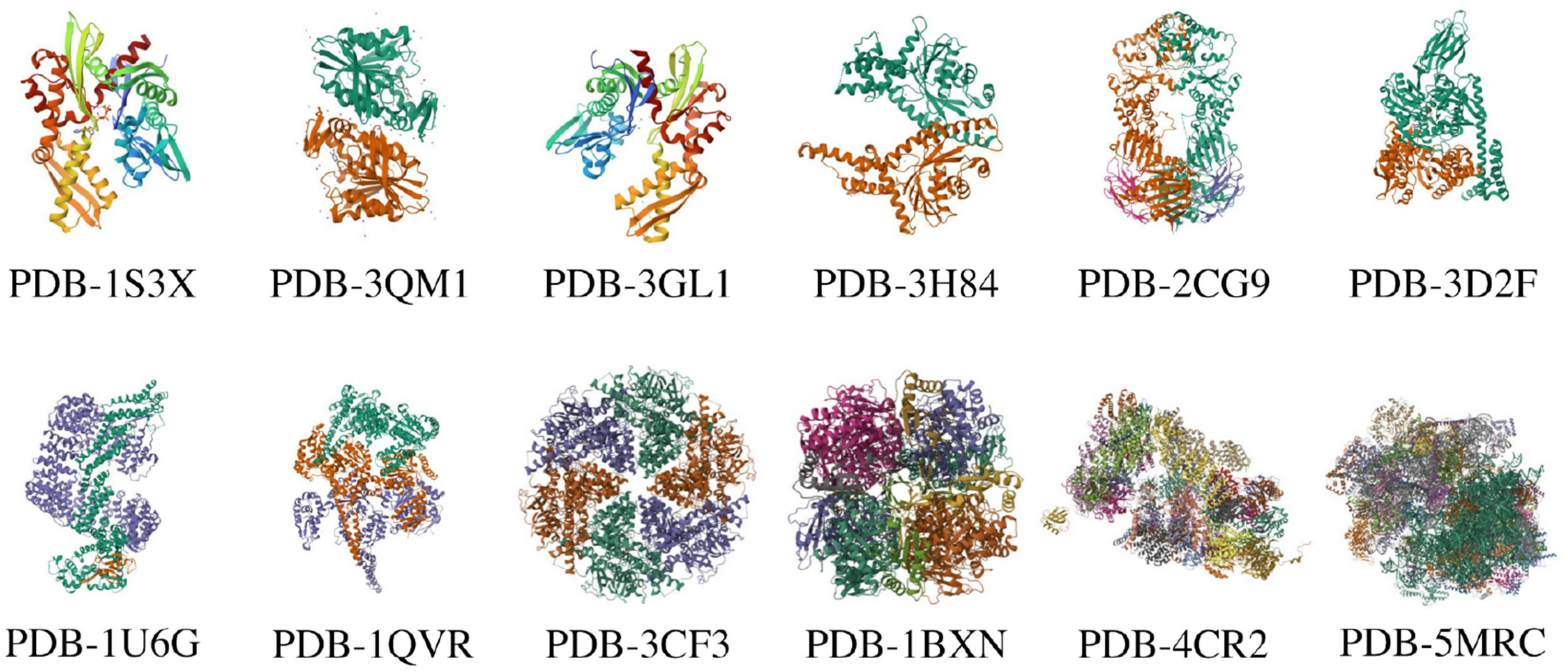
The macromolecule classes of SHREC19 and SHREC21. For detailed descriptions of each class, please refer to the original publications: SHREC19 (Gubins *et al.* 2019) and SHREC21 (Gubins *et al.* 2021).

*Dataset setting.* We use three datasets in our experiments: SHREC19 (Gubins *et al.* 2019), SHREC21 (Gubins *et al.* 2021) (see Fig. 5), and a real dataset from the CryoET Data Portal. SHREC19 has 12 macromolecule classes and 20 785 samples, SHREC21 has 13 classes and 16 291 samples, and the real dataset includes 12 classes and 1857 samples. Tomograms were segmented into 32×32×32 subtomograms using ground-truth annotations. For SHREC19 and SHREC21, subtomograms from the first 9 tomograms were used for training, with the 10th tomogram as the ID test set. A similar partition was applied to the real dataset. For testing, we created six test sets with both ID and OOD data, denoted as “ID&OOD”: “SHREC19&SHREC21,” “SHREC19&Real,” “SHREC21&SHREC19,”“SHREC21&Real,”“Real&SHREC19,” and “Real&SHREC21.”

*Evaluation metrics.* We evaluate our method using three metrics: (1) FPR95: the false positive rate of OOD samples at a 95% true positive rate for ID samples, (2) AUROC: the area under the receiver operating characteristic curve, and (3) ACC: the multi-class classification accuracy.

### 3.2 The effectiveness of our subtomogram classification framework

To validate the effectiveness of our proposed framework, We incorporated the original classification method [i.e. CB3D (Che *et al.* 2018), DSRF3D-v2 (Che *et al.* 2018), RB3D (Che *et al.* 2018), and CFN (Wang *et al.* 2024)], into our framework, enabling it with OOD detection capabilities, and conducted comparative experiments on the constructed test set containing OOD data.

The results in Table 1 show that methods with OOD detection outperform their original counterparts. For instance, CB3D achieved 40.78% and 38.06% accuracy on the “SHREC19&SHREC21” and “SHREC19&Real” datasets, respectively. With our framework, accuracy increased to 66.94% and 62.38%, improving by 26.16% and 24.32%. For DSRF3D-v2, classification accuracy on the “Real&SHREC19” and “Real&SHREC21” datasets was initially 4.26% and 5.51%, but with OOD detection, it improved to 65.88% and 89.44%. This highlights our framework’s ability to handle unknown class data, with more significant improvements as unknown data increase. Additionally, integrating OOD detection with Soft LMCCL (Du *et al.* 2019) showed similar performance gains. Overall, our framework effectively enhances existing methods’ ability to manage unknown class data.

**Table 1.** The comparative accuracy results of the subtomogram classification method with and without OOD detection.^a^

Methods	SHREC19&SHREC21	SHREC19&Real	SHREC21&SHREC19	SHREC21&Real	Real&SHREC19	Real&SHREC21
CB3D	40.78%	38.06%	33.09%	35.55%	2.78%	3.60%
CB3D+OOD	**66.94%** ^b^	**62.38%**	**62.48%**	**65.50%**	**91.83%**	**89.44%**
DSRF3D-v2	33.93%	38.34%	34.06%	36.59%	4.26%	5.51%
DSRF3D-v2+OOD	**63.62%**	**64.45%**	**51.39%**	**56.18%**	**65.88%**	**89.44%**
RB3D	32.42%	36.93%	32.17%	34.56%	6.61%	8.54%
RB3D+OOD	**58.03%**	**59.53%**	**51.66%**	**59.29%**	**41.55%**	**71.85%**
CFN	32.14%	36.67%	39.38%	37.67%	6.64%	8.59%
CFN+OOD	**40.86%**	**37.95%**	**39.43%**	**41.22%**	**29.25%**	**31.27%**
Soft LMCCL	31.68%	36.25%	31.84%	34.20%	4.74%	6.13%
Soft LMCCL+OOD	**76.70%**	**63.40%**	**61.80%**	**64.90%**	**92.90%**	**93.90%**

a “ID&OOD” denotes the source of ID data and OOD data.

b The optimal values in this table are in **bold**.

### 3.3 Comparison with SOTA methods

To evaluate the proposed noise-robust method, we conduct comparative experiments against other SOTA methods with OOD detection within our framework.

The results are presented in Table 2, with additional results provided in the Supplementary Material. In Table 2, all the compared SOTA methods include the OOD framework and are equipped with OOD detection to ensure the fairness in the comparison. We used FPR95 and AUROC as the metrics for OOD detection. A lower FPR95 and higher AUROC indicate stronger OOD detection capability. As shown in Table 2, our method achieves a smaller FPR95 and higher AUROC across all datasets compared to other methods. For example, in Table 2, CFN achieves an FPR95 of 55.26% and RB3D attains an AUROC of 87.40% on the “SHREC19&SHREC21” dataset. In contrast, our method achieves an FPR95 of 0 and an AUROC of 99.99%. At the same time, we use the constructed dataset containing both ID and OOD data to evaluate the classification performance of these methods. Our method achieves higher accuracy on these test sets compared to other methods. The experimental results in Table 2 demonstrate that our method outperforms other methods in both OOD detection and classification performance.

**Table 2.** The comparative experimental results between our method and other SOTA methods with OOD detection within our framework on “SHREC19&SHREC21” and “SHREC21&SHREC19.”^a^

	SHREC19&SHREC21			SHREC21&SHREC19		
Methods	FPR95 ↓	AUROC ↑	ACC ↑	FPR95 ↓	AUROC ↑	ACC ↑
CB3D+OOD	91.52%	68.27%	66.94%	88.31%	55.31%	62.48%
DSRF3D-v2+OOD	71.80%	82.48%	63.62%	88.26%	58.18%	51.39%
RB3D+OOD	62.12%	87.40%	58.03%	94.23%	51.46%	51.66%
CFN+OOD	55.26%	57.01%	40.86%	90.25%	67.99%	39.43%
Soft LMCCL+OOD	91.10%	46.20%	76.70%	54.80%	87.80%	61.80%
Ours	**0.00%** ^b^	**99.99%**	**83.47%**	**0.05%**	**99.86%**	**90.87%**

a Metrics include FPR95, AUROC, and ACC.

b The optimal values in this table are in **bold**.

### 3.4 Visualization comparison

To further evaluate the feature modeling and OOD detection capability of our method, we present the comparison between our method and other methods in terms of t-SNE visualization and score distribution.

**Figure 6.**
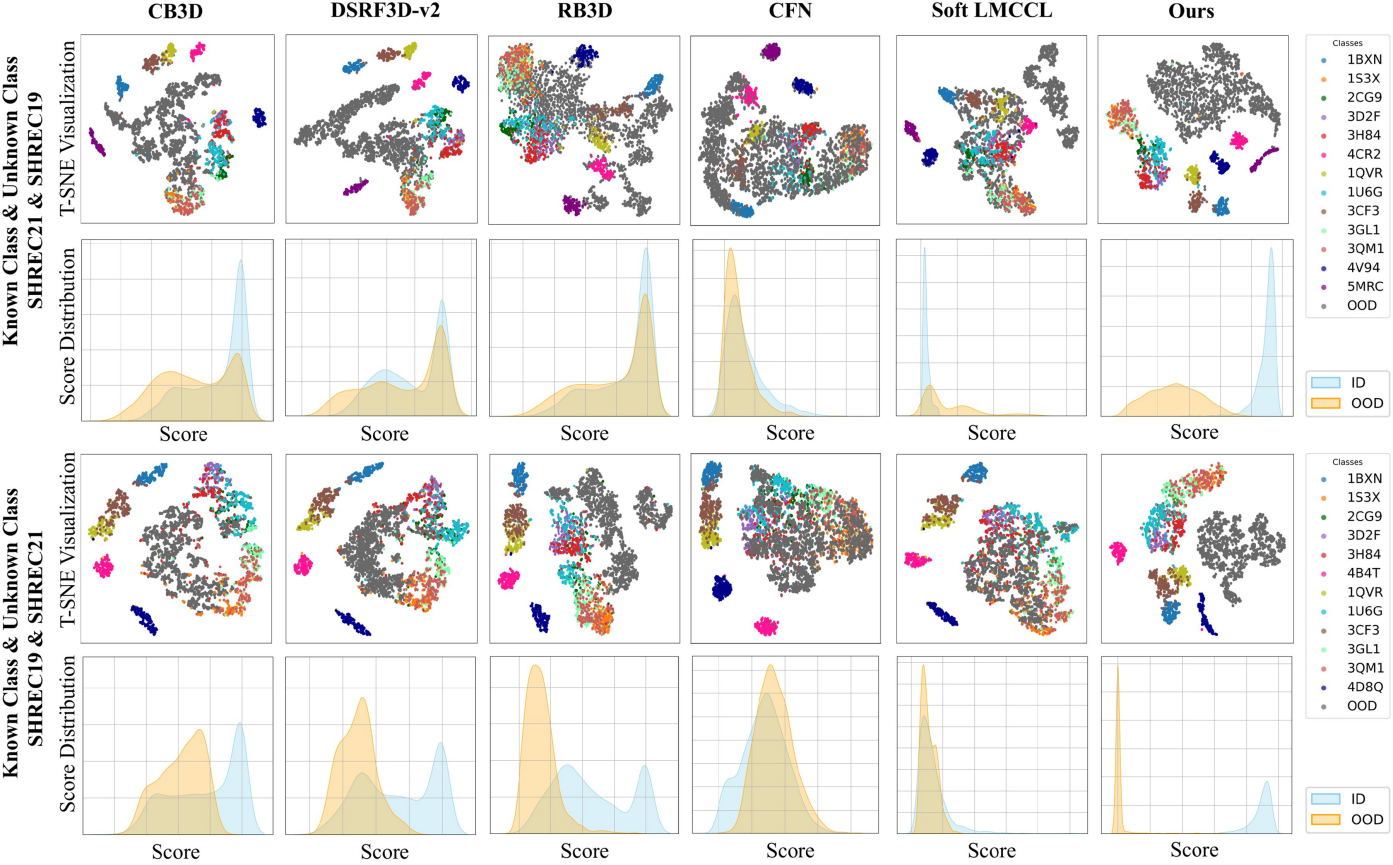
The comparative results of t-SNE visualization and score distribution on the “SHREC19&SHREC21” and “SHREC21&SHREC19” datasets. CB3D, DSRF3D-v2, RB3D, and CFN use maximum softmax probability-based (MSP) score (Hendrycks and Gimpel 2016), Soft LMCCL uses the L2 distance-based score (Du *et al.* 2019), and our method uses the Mahalanobis distance-based score [see Equation (20)].

The results are presented in Fig. 6 (More visualization results are in Supplementary Material.). In the t-SNE visualization, the OOD feature distribution of our method is compact and well-separated from the ID features. For example, on the “SHREC21&SHREC19” dataset, the t-SNE visualization of our method shows clear boundaries between OOD and ID features, with the compact OOD features. In contrast, the OOD features of CFN, RB3D, and Soft LMCCL overlap with their ID features. This indicates that our method has stronger representation capability, effectively enhancing model performance through the construction of a more discriminative embedding space. Our method’s ID and OOD score distributions are well-separated. For example, on the “SHREC19&SHREC21” dataset, our OOD score distribution does not have overlap with the ID score distribution. In comparison, other methods exhibit a larger overlap between the ID score distribution and the OOD score distribution. The visualization results clearly demonstrate the effectiveness of our method in feature modeling and OOD detection.

### 3.5 Ablation experiments

In this section, we present ablation experiments for the critical modules of our method, including 3D DWT-based encoder, Mahalanobis distance-based OOD detector, and the adaptive classifier.

*The effectiveness of 3D DWT-based encoder.* To evaluate the effectiveness of the 3D DWT-based encoder, we compared it with an encoder without DWT and with DWT-based encoders using different wavelets (see Table 3). The results show that the 3D DWT-based encoder outperforms the original 3D ResNet18. For instance, the original 3D ResNet18 achieved only 43.24% accuracy on the “SHREC19&SHREC21” dataset and 41.53% on the “SHREC21&SHREC19” dataset, while the 3D DWT-based encoder with Haar wavelets improved accuracy by 39.02% and 48.00%, respectively. These results demonstrate the encoder’s effectiveness. Additionally, experiments with different wavelets revealed that the third-order Cohen wavelet (ch3.3) achieved the best OOD detection and classification performance in most cases, leading us to select it for our method.

**Table 3.** The comparative experimental results involving an encoder without DWT and DWT-based encoders using different wavelets on the “SHREC19&SHREC21” and “SHREC21&SHREC19” datasets.^a^

Encoder		SHREC19&SHREC21			SHREC21&SHREC19		
		FPR95 ↓	AUROC ↑	ACC ↑	FPR95 ↓	AUROC ↑	ACC ↑
3D Resnet18		0.00%	99.99%	43.24%	0.62%	99.65%	41.53%
3D DWT-based Resnet18	haar	0.00%	99.99%	82.26%	1.70%	99.06%	89.53%
	ch2.2	0.00%	99.99%	82.96%	0.94%	99.26%	89.82%
	ch3.3	**0.00%** ^b^	**99.99%**	83.47%	**0.05%**	**99.86%**	**90.87%**
	ch4.4	0.00%	99.87%	**83.82%**	2.79%	99.36%	88.74%
	ch5.5	0.63%	99.86%	82.42%	3.79%	99.17%	88.12%

a “haar” represents Haar wavelet (Daubechies 1992). “ch2.2,” “ch3.3,” “ch4.4,” and “ch5.5” represent Cohen wavelet (Cohen *et al.* 1992) with 2 to 5 orders.

b The optimal values in this table are in **bold**.

*The effectiveness of mahalanobis distance-based OOD detector.* To validate the effectiveness of the Mahalanobis distance-based OOD Detector, we performed ablation experiments using different OOD detectors (see Table 4). From Table 4, it can be observed that our OOD detector achieves the highest classification accuracy, the lowest FPR95, and the highest AUROC across various datasets. For the “SHREC21&SHREC19” dataset, the KNN method achieved an FPR95 of 1.80%, an AUROC of 99.41%, and a classification accuracy of 89.55%. In contrast, our OOD detector achieved an FPR95 of 0.05%, an AUROC of 99.86%, and a classification accuracy of 90.87%. This represents a 1.75% reduction in FPR95, a 0.45% increase in AUROC, and a 1.32% improvement in classification accuracy compared to the KNN method. On the “SHREC19&SHREC21” dataset, compared to the VIM method, our OOD detector improved classification accuracy by 0.21%. The experimental results demonstrate our Mahalanobis distance-based OOD detector outperforms other OOD detectors.

**Table 4.** The comparative experimental results of different OOD detectors on the “SHREC19&SHREC21” and “SHREC21&SHREC19” datasets.^a^

	SHREC19&SHREC21			SHREC21&SHREC19		
OOD Detector	FPR95 ↓	AUROC ↑	ACC ↑	FPR95 ↓	AUROC ↑	ACC ↑
MSP	99.50%	54.47%	41.10%	68.99%	80.87%	52.12%
Energy	99.25%	36.12%	40.91%	51.37%	88.44%	61.73%
Maxlogit	99.12%	36.63%	40.94%	50.24%	88.18%	62.54%
VIM	0.00%	99.98%	83.26%	33.43%	96.19%	73.32%
NNGuide	91.95%	59.53%	44.40%	72.06%	80.39%	49.80%
KNN	1.57%	99.61%	83.15%	1.80%	99.41%	89.55%
Ours	**0.00%** ^b^	**99.99%**	**83.47%**	**0.05%**	**99.86%**	**90.87%**

a Detailed descriptions of the OOD detectors and corresponding references can be found in the Supplementary Materials.

b The optimal values in this table are in **bold**.

*The effectiveness of the adaptive classifier.* To validate the effectiveness of the adaptive classifier, we conducted comparative experiments between the cosine similarity-based classifier, t-vMF similarity-based classifier, and our adaptive classifier (see Table 5). The results in Table 5 show that our adaptive classifier achieves the lowest FPR95, the highest AUROC, and the highest classification accuracy across different data scales. On the “SHREC19&SHREC21” dataset, the adaptive classifier using cosine similarity achieved an accuracy of 83.47%, FPR95 of 0, and AUROC of 99.99%. The t-vMF similarity-based classifier achieved an accuracy of 74.56%, FPR95 of 18.35%, and AUROC of 96.12%. When the training set size is sufficiently large, our adaptive classifier also used cosine similarity and outperformed the t-vMF similarity-based classifier. On the “Real&SHREC19” and “Real&SHREC21” datasets, the adaptive classifier using t-vMF similarity achieved 98.09% and 97.36% accuracy with almost zero FPR95, respectively. The cosine similarity-based classifier achieved only 27.97% and 64.85% accuracy. The experimental results demonstrate that our adaptive classifier achieves higher classification accuracy and better OOD detection performance on datasets with varying scales.

**Table 5.** The comparative experimental results of the classifier based on different similarity.^a^

	Cosine Similarity-Based Classifier			t-vMF Similarity-Based Classifier			Adaptive Classifier		
Dataset	FPR95 ↓	AUROC ↑	ACC ↑	FPR95 ↓	AUROC ↑	ACC ↑	FPR95 ↓	AUROC ↑	ACC ↑
SHREC19&SHREC21	0.00%	99.99%	83.47%	18.35%	96.12%	74.56%	**0.00%** ^b^	**99.99%**	**83.47%**
SHREC19&Real	5.50%	98.90%	82.81%	28.02%	94.84%	70.54%	**5.50%**	**98.90%**	**82.81%**
SHREC21&SHREC19	0.05%	99.86%	90.87%	3.69%	98.92%	87.87%	**0.05%**	**99.86%**	**90.87%**
SHREC21&Real	2.42%	99.45%	88.83%	9.48%	98.06%	84.13%	**2.42%**	**99.45%**	**88.83%**
Real&SHREC19	77.89%	90.18%	27.97%	0.00%	99.99%	98.09%	**0.00%**	**99.99%**	**98.09%**
Real&SHREC21	40.41%	94.15%	64.85%	0.25%	99.92%	97.36%	**0.25%**	**99.92%**	**97.36%**

a Metrics include FPR95, AUROC, and ACC.

b The optimal values in this table are in **bold**.

## 4 Conclusion

Current cryo-ET subtomogram classification methods often misclassify OOD data. To address this, we propose a subtomogram classification method combining a 3D wavelet transform-based encoder for noise-robust feature extraction and a Mahalanobis distance-based OOD detector, along with an adaptive classifier for varying dataset scales. We constructed a test set with both ID and OOD data to evaluate our method. Experimental and visualization results show that our method outperforms others in OOD detection and classification. Ablation experiments confirmed the importance of key modules.

Although our method primarily focuses on the classification of macromolecules with distinct structural differences and demonstrates high performance in distinguishing such macro- molecules, it is a challenging and promising direction to extend our method to classify macromolecules with minor structural differences (e.g. different conformations of the same structure) (Powell and Davis 2024, Rangan *et al.* 2024). Classifying different conformations of the same structure will be a key focus of our future work.

## Supplementary Material

btaf274_Supplementary_Data

## Data Availability

The real data underlying this article are available in CryoET Data Portal at https://cryoetdataportal.czscience.com, and can be accessed with DS-10000, DS-10004, DS-10008, DS-10009, DS-10301, DS-10439. The public dataset SHREC19 and SHREC21 are available at https://doi.org/10.34894/XSKKQV and https://doi.org/10.34894/XRTJMA, respectively.
