# Supplementary material for: A noise-robust classification method for cryo-ET subtomograms with out-of-distribution detection
Source: Bioinformatics. 2025 May 13;41(5):btaf274. doi: 10.1093/bioinformatics/btaf274 (PMC12106275; doi:10.1093/bioinformatics/btaf274)
Supplement: btaf274_Supplementary_Data [file btaf274_supplementary_data.pdf]

Supplementary Material for “A Noise-Robust Classification Method for Cryo-ET Subtomograms with Out-of-Distribution Detection”

Our supplementary materials contain extensive additional results, including: comprehensive comparisons with state-of-the-art (SOTA) methods, the visualization comparison, the ablation study of 3D DWT-based encoder, and the effectiveness of Mahalanobis distance-based OOD detector. Furthermore, we provide a detailed descriptions of the wavelet filters employed in our framework, along with references of all OOD detectors evaluated in this work.

Comparison with SOTA Methods

To evaluate the proposed noise-robust method, we conduct comparative experiments against other state-of-the-art (SOTA) methods with OOD detection within our framework. The results are presented in Table S1 and S2.

Table S1. The comparative experimental results between our method and other SOTA methods with OOD detection within our framework on “SHREC19&Real” and “SHREC21&Real”.

	SHREC19&Real			SHREC21&Real		
Methods	FPR95 ↓	AUROC ↑	ACC ↑	FPR95 ↓	AUROC ↑	ACC ↑
CB3D+OOD	92.89%	72.13%	62.38%	75.57%	65.29%	65.50%
DSRF3D-v2+OOD	48.68%	83.72%	64.45%	76.66%	71.34%	56.18%
RB3D+OOD	50.86%	79.56%	59.53%	79.70%	65.10%	59.29%
CFN+OOD	95.25%	53.78%	37.95%	64.89%	91.19%	41.22%
Soft LMCCL+OOD	81.70%	66.50%	63.40%	60.30%	78.30%	64.90%
Ours	**5.50%**	**98.90%**	**82.81%**	**2.42%**	**99.45%**	**88.83%**

Table S2. The comparative experimental results between our method and other SOTA methods with OOD detection within our framework on “Real&SHREC19” and “Real&SHREC21”.

	Real&SHREC19			Real&SHREC21		
Methods	FPR95 ↓	AUROC ↑	ACC ↑	FPR95 ↓	AUROC ↑	ACC ↑
CB3D+OOD	100%	8.61%	91.83%	82.79%	88.54%	89.44%
DSRF3D-v2+OOD	98.96%	24.62%	65.88%	83.54%	61.34%	89.44%
RB3D+OOD	99.43%	47.93%	41.55%	64.76%	77.91%	71.85%
CFN+OOD	72.87%	82.33%	29.25%	76.49%	76.65%	31.27%
Soft LMCCL+OOD	43.10%	88.30%	92.90%	83.50%	47.50%	93.90%
Ours	**0.00%**	**99.99%**	**98.09%**	**0.25%**	**99.92%**	**97.36%**

Visualization Comparison

To further demonstrate the visualization results of our method, we present the t-SNE visualization and score distribution on additional datasets in Fig. S1 and Fig. S2.

In Fig. S1, on the “Real&SHREC21” dataset, the t-SNE visualization of our method shows clear boundaries between OOD and ID features, with the compact OOD feature. Our OOD score distribution has no overlap with the ID score distribution. A similar performance of our method is observed in Fig. S2 for the “Real&SHREC19” dataset. In Fig. S2, for the ”SHREC19&Real” dataset, the OOD features of our method exhibit a clear boundary with the ID features in the t-SNE visualization. Our OOD score distribution has a small overlap with the ID score distribution. In contrast, other methods show larger overlap between their OOD score distribution and ID score distribution. These results validated the effectiveness of our method across different datasets.

The Effectiveness of 3D DWT-Based Encoder

To further validate the effectiveness of the 3D DWT-based encoder, we compared the results with and without the 3D DWT-based encoder on more datasets. We also evaluated the performance of our 3D DWT-based encoder using different wavelet.

We provided the experimental results in Table S3 and Table S4. The results show that the 3D DWT-based ResNet18 achieves better classification performance compared to the original 3D ResNet18. For example, the 3D DWT ResNet18 with Haar wavelets improved the classification accuracy of the original one by 7.76% on “Real&SHREC19” dataset. The results demonstrate that our 3D DWT-based encoder is effective. Additionally, we conducted comparative experiments on 3D DWT-based ResNet18 with different wavelets. According to the experimental results, the 3D DWT ResNet18 based on the third-order Cohen wavelet (ch3.3) achieved the best OOD detection and classification performance in most cases. Based on these results, we selected the 3D DWT-based encoder based on the third-order Cohen wavelet as the encoder for our method.

The Effectiveness of the Mahalanobis Distance-Based OOD Detector

To further validate the effectiveness of the Mahalanobis distance-based OOD Detector, we performed experiments by comparing different OOD detectors on additional datasets, as shown in Table S5 and Table S6.

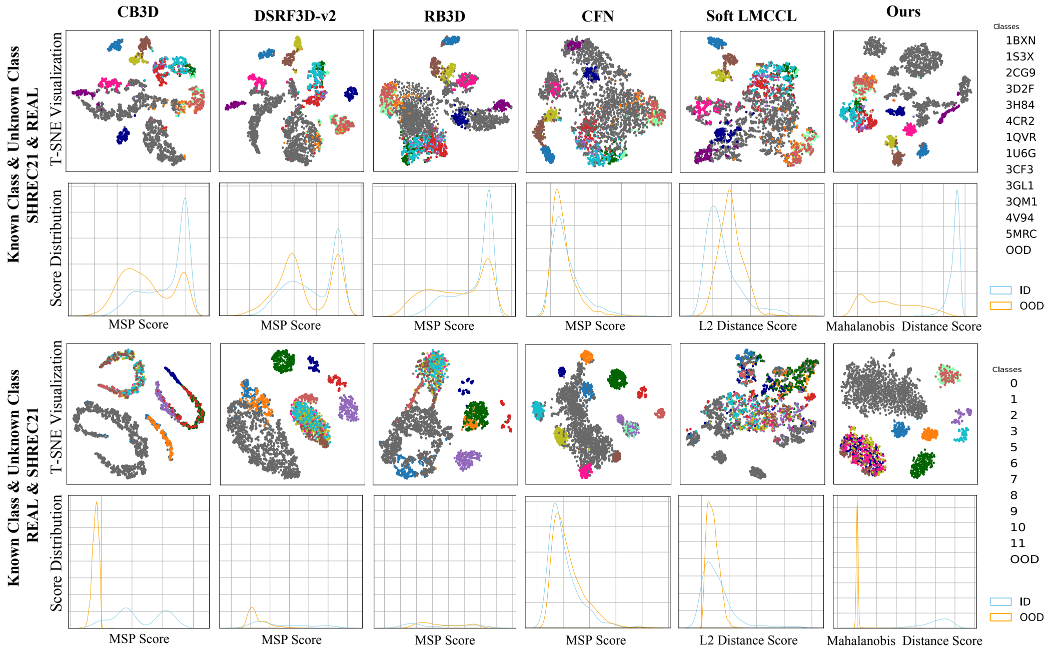

Fig. S1. The comparative results of t-SNE visualization and score distribution on the "Real&SHREC21" and "SHREC21&Real" datasets.

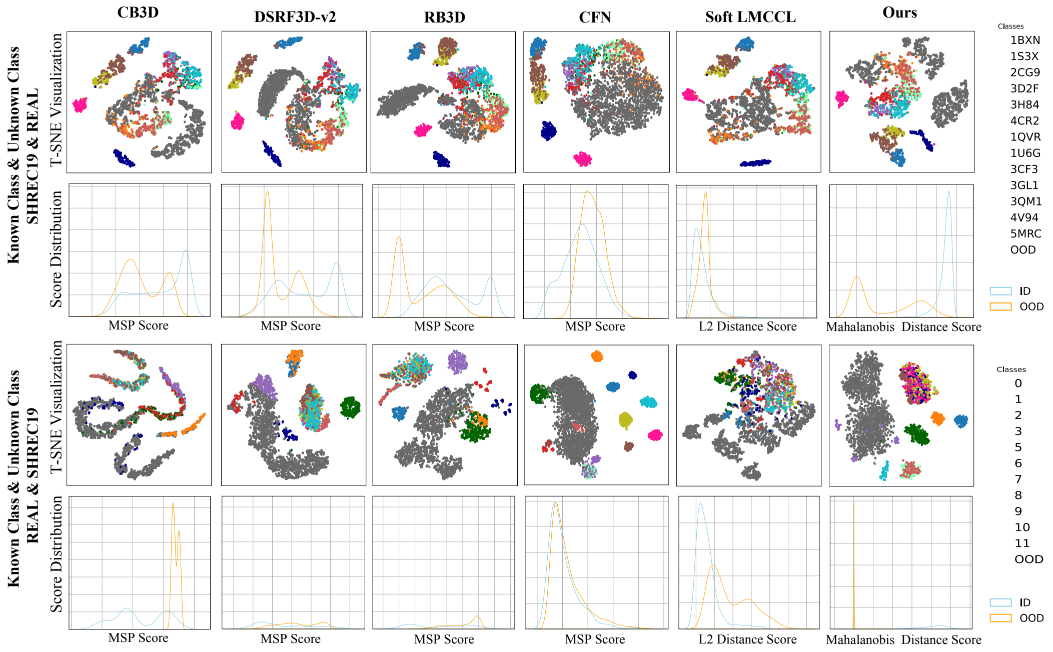

Fig. S2. The comparative results of t-SNE visualization and score distribution on the "Real&SHREC19" and "SHREC19&Real" datasets.

The results indicate that the Mahalanobis distance-based OOD detector achieves the best classification accuracy, the lowest FPR95, and the highest AUROC on different datasets. For instance, on the "Real&SHREC21" dataset, the KNN method achieved an FPR95 of 2.07%, an AUROC of 99.35%, and a classification accuracy of 95.73%. In comparison, the Mahalanobis distance-based OOD detector achieved an FPR95 of 0.25%, an AUROC of 99.92%, and a classification accuracy of 97.36%. This corresponds to a 1.82% reduction in FPR95, a 0.57% increase in AUROC, and a 1.63% improvement in classification accuracy over the KNN method. The experimental results demonstrate that our Mahalanobis distance-based OOD detector outperforms other OOD detectors.

The Impact of the Threshold Parameter λ

To investigate the impact of the threshold parameter λ, we conducted the experiment within a reasonable range of λ on the "SHREC19&SHREC21" dataset. The results are shown in Fig. S3 and the results show that as λ increases, the classification

Table S3. The comparative experimental results involving an encoder without DWT and DWT-based encoders using different wavelets on the “Real&SHREC21” and “SHREC21&Real” datasets.

Encoder		SHREC21&Real			Real&SHREC21		
		FPR95 ↓	AUROC ↑	ACC ↑	FPR95 ↓	AUROC ↑	ACC ↑
3D Resnet18		3.45%	99.54%	35.59%	8.05%	96.43%	96.12%
3D DWT-based Resnet18	haar	3.83%	99.40%	88.40%	9.68%	97.23%	96.12%
	ch2.2	**1.34%**	98.83%	**89.15%**	1.35%	98.83%	81.50%
	ch3.3	2.42%	99.45%	88.83%	**0.25%**	**99.92%**	**97.36%**
	ch4.4	6.20%	98.85%	86.48%	5.97%	98.58%	92.91%
	ch5.5	7.81%	**99.65%**	84.97%	28.72%	98.58%	91.91%

Table S4. The comparative experimental results involving an encoder without DWT and DWT-based encoders using different wavelets on the “Real&SHREC19” and “SHREC19&Real” datasets.

Encoder		Real&SHREC19			SHREC19&Real		
		FPR95 ↓	AUROC ↑	ACC ↑	FPR95 ↓	AUROC ↑	ACC ↑
3D Resnet18		0.33%	99.24%	90.27%	9.21%	98.14%	80.69%
3D DWT-based Resnet18	haar	0.00%	99.87%	98.03%	**5.44%**	98.76%	80.70%
	ch2.2	29.50%	92.20%	69.86%	12.18%	97.63%	78.33%
	ch3.3	**0.00%**	**99.99%**	**98.09%**	5.50%	**98.90%**	**82.81%**
	ch4.4	0.00%	99.88%	98.00%	7.65%	98.10%	81.28%
	ch5.5	0.57%	99.86%	76.55%	12.93%	97.17%	77.72%

Table S5. The comparative experimental results of different OOD detectors on the “Real&SHREC21” and “SHREC21&Real” datasets.

	Real&SHREC21			SHREC21&Real		
OOD Detector	FPR95 ↓	AUROC ↑	ACC ↑	FPR95 ↓	AUROC ↑	ACC ↑
MSP	73.41%	57.22%	32.11%	92.13%	55.01%	41.2%
Energy	43.49%	88.67%	58.89%	83.84%	60.12%	45.34%
Maxlogit	61.85%	62.71%	42.40%	84.75%	59.61%	44.94%
VIM	100%	0.79%	8.04%	14.87%	97.24%	81.55%
NNGuide	46.45%	86.40%	55.96%	84.21%	63.41%	45.14%
KNN	2.07%	99.35%	95.73%	23.17%	86.63%	77.84%
Ours	**0.25%**	**99.92%**	**97.36%**	**2.42%**	**99.45%**	**88.83%**

Table S6. The comparative experimental results of different OOD detectors on the “Real&SHREC19” and “SHREC19&Real” datasets.

	Real&SHREC19			SHREC19&Real		
OOD Detector	FPR95 ↓	AUROC ↑	ACC ↑	FPR95 ↓	AUROC ↑	ACC ↑
MSP	21.64%	92.26%	78.42%	97.09%	51.99%	39.47%
Energy	7.20%	98.42%	91.69%	92.56%	48.27%	41.36%
Maxlogit	96.02%	9.71%	89.34%	93.43%	48.44%	30.47%
VIM	94.37%	40.29%	11.53%	28.50%	92.99%	70.69%
NNGuide	6.91%	98.42%	91.73%	96.17%	66.30%	39.67%
KNN	0.19%	99.94%	97.95%	14.49%	96.35%	77.74%
Ours	**0.00%**	**99.99%**	**98.09%**	**5.50%**	**98.90%**	**82.81%**

accuracy continuously improves, reaching the highest accuracy at $\lambda = 95\%$. This result indicates that the λ value (95%) in our manuscript is reasonable.

Comparison of Time Costs

To investigate the time cost of our method, we present the time consumption of all methods on the typical “SHREC19&SHREC21” dataset in Table S7. The results demonstrate that our method has the lowest time consumption.

Table S7. Comparison of time costs with and without OOD detection on “SHREC19&SHREC21” dataset.

Model	Latency (s)	
	without OOD	with OOD
CB3D	20.8471	24.9806
DSRF3D-v2	20.7867	21.7350
RB3D	26.0286	26.7724
CFN	32.5665	33.7662
Soft LMCCL	29.1020	26.6535
Ours	**11.4385**	**13.4049**

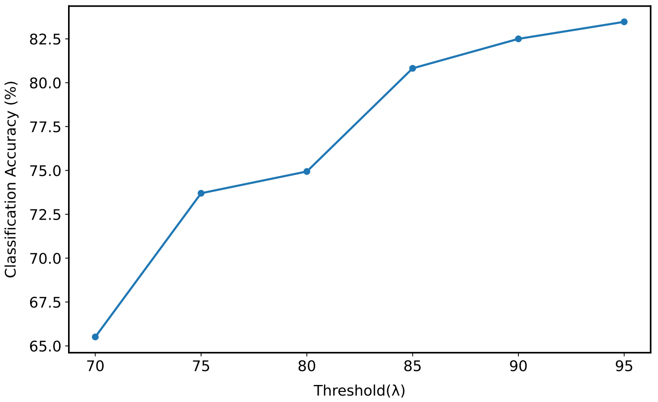

Fig. S3. The experimental results for different threshold parameter λ "SHREC19&SHREC21" dataset.

The Impact of the Training Set Size

We conducted ablation experiments to evaluate the performance of each classifier across different scales on the representative "SHREC19&SHREC21" dataset. The experimental results in Fig. S4 show that the cosine similarity-based classifier performs better with larger training sets, while the t-vMF similarity-based classifier excels with smaller training sets, aligning with our experimental setup.

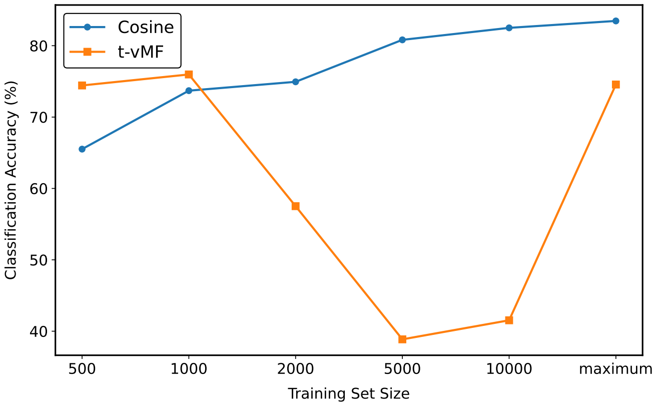

Fig. S4. The ablation experimental results for the training set size on "SHREC19&SHREC21" dataset.

The Filters of the Cohen Wavelet

The proposed 3D DWT-based encoder uses Cohen wavelets (Cohen et al., 1992). For clarity, we provide the values of the low-pass filters l and the dual low-pass filters $\tilde{l}$ in Table S8. The values in the high-pass filters h can be obtained by the following formulation:

$$h_k = (-1)^k \tilde{l}_{N-k}, \tag{S1}$$

where N is an odd number.

Detailed Descriptions of the OOD Detectors

To validate the effectiveness of the Mahalanobis distance-based OOD Detector, we performed ablation experiments using different OOD detectors: MSP (Hendrycks and Gimpel, 2016), Energy (Liu et al., 2020), Maxlogit (Zhang and Xiang, 2023), VIM (Wang et al., 2022), NNGuide (Park et al., 2023), and KNN (Sun et al., 2022). Detailed implementations and theoretical foundations of these methods are provided in their respective publications.

Table S8. The low-pass filters of Cohen wavelet. p and $\tilde{p}$ are the order parameters of Cohen wavelet. For the filters of the given order in the table, all undefined filter values are set to 0.

$(p,\tilde{p})$		(2, 2)		(3,3)		(4,4)		(5,5)	
filter	k	$\mathbf{l}$	$\tilde{\mathbf{l}}$	$\mathbf{l}$	$\tilde{\mathbf{l}}$	$\mathbf{l}$	$\tilde{\mathbf{l}}$	$\mathbf{l}$	$\tilde{\mathbf{l}}$
	-5							0.01346	0.00000
	-4					0.00000	0.00000	-0.00269	0.00000
	-3			0.00000	0.06629	-0.06454	0.03783	-0.13671	0.03969
	-2	0.00000	0.00000	0.00000	-0.19887	-0.04069	-0.02385	-0.09350	0.00795
	-1	0.35355	-0.17678	0.17678	-0.15468	0.41809	-0.11062	0.47680	-0.05446
l_k	0	0.70711	0.35355	0.53033	0.99437	0.78849	0.37740	0.89951	0.34561
	1	0.35355	1.06066	0.53033	0.99437	0.41809	0.85270	0.47680	0.73666
	2	0.00000	0.35355	0.17678	-0.15468	-0.04069	0.37740	-0.09350	0.34561
	3	0.00000	-0.17678	0.00000	-0.19887	-0.06454	-0.11062	-0.13671	-0.05446
	4			0.00000	0.06629	0.00000	-0.02385	-0.00269	0.00795
	5					0.00000	0.03783	0.01346	0.03969

Details Descriptions of the Real Dataset

We used the datasets from CryoET Data Portal[1] and the ID of these datasets are: DS-10000 (de Teresa-Trueba et al., 2023), DS-10004 (Schiøtz et al., 2024), DS-10008 (Dietrich et al., 2022), DS-10009 (Van den Hoek et al., 2022), DS-10301 (Khavnekar et al., 2023), and DS-10439 (Martinez-Sanchez et al., 2024). We have added the details of real dataset in Table S9 of the Supplementary Material.

Table S9. The details of the real dataset.

Dataset Name	Acknowledgement
DS-10000	https://doi.org/10.1038/s41592-022-01746-2
DS-10004	https://doi.org/10.1038/s41592-023-02113-5
DS-10008	https://doi.org/10.1038/s41586-022-04971-z
DS-10009	https://doi.org/10.1126/science.abm6704
DS-10301	https://doi.org/10.1093/micmic/ozad067.480
DS-10439	https://doi.org/10.1109/TMI.2024.3398401

References

Cohen, A., I. Daubechies, and J.-C. Feauveau (1992). Biorthogonal bases of compactly supported wavelets. *Communications on pure and applied mathematics 45*(5), 485–560.

de Teresa-Trueba, I., S. K. Goetz, A. Mattausch, F. Stojanovska, C. E. Zimmerli, M. Toro-Nahuelpan, D. W. Cheng, F. Tollervey, C. Pape, M. Beck, et al. (2023). Convolutional networks for supervised mining of molecular patterns within cellular context. *Nature Methods 20*(2), 284–294.

Dietrich, H. M., R. D. Righetto, A. Kumar, W. Wietrzynski, R. Trischler, S. K. Schuller, J. Wagner, F. M. Schwarz, B. D. Engel, V. Müller, et al. (2022). Membrane-anchored hdcr nanowires drive hydrogen-powered co2 fixation. *Nature 607*(7920), 823–830.

Hendrycks, D. and K. Gimpel (2016). A baseline for detecting misclassified and out-of-distribution examples in neural networks. *arXiv preprint arXiv:1610.02136*.

Khavnekar, S., R. Kelley, F. Waltz, W. Wietrzynski, X. Zhang, M. Obr, G. Tagiltsev, F. Beck, W. Wan, J. Briggs, et al. (2023). Towards the visual proteomics of c. reinhardtii using high-throughput collaborative in situ cryo-et.

Liu, W., X. Wang, J. Owens, and Y. Li (2020). Energy-based out-of-distribution detection. *Advances in neural information processing systems 33*, 21464–21475.

Martinez-Sanchez, A., L. Lamm, M. Jasnin, and H. Phelippeau (2024). Simulating the cellular context in synthetic datasets for cryo-electron tomography. *IEEE Transactions on Medical Imaging*.

Park, J., Y. G. Jung, and A. B. J. Teoh (2023). Nearest neighbor guidance for out-of-distribution detection. In *Proceedings of the IEEE/CVF International Conference on Computer Vision*, pp. 1686–1695.

Schiøtz, O. H., C. J. Kaiser, S. Klumpe, D. R. Morado, M. Poege, J. Schneider, F. Beck, D. P. Klebl, C. Thompson, and J. M. Plitzko (2024). Serial lift-out: sampling the molecular anatomy of whole organisms. *Nature Methods 21*(9), 1684–1692.

Sun, Y., Y. Ming, X. Zhu, and Y. Li (2022). Out-of-distribution detection with deep nearest neighbors. In *International Conference on Machine Learning*, pp. 20827–20840. PMLR.

Van den Hoek, H., N. Klena, M. A. Jordan, G. Alvarez Viar, R. D. Righetto, M. Schaffer, P. S. Erdmann, W. Wan, S. Geimer, J. M. Plitzko, et al. (2022). In situ architecture of the ciliary base reveals the stepwise assembly of intraflagellar transport trains. *Science 377*(6605), 543–548.

Wang, H., Z. Li, L. Feng, and W. Zhang (2022). Vim: Out-of-distribution with virtual-logit matching. In *Proceedings of the IEEE/CVF conference on computer vision and pattern recognition*, pp. 4921–4930.

Zhang, Z. and X. Xiang (2023). Decoupling maxlogit for out-of-distribution detection. In *Proceedings of the IEEE/CVF Conference on Computer Vision and Pattern Recognition*, pp. 3388–3397.

[1] https://cryoetdataportal.czscience.com
